# Coronal lumbar horizontal shift and lower limb force line in patients with knee osteoarthritis: a correlation study

**DOI:** 10.3389/fbioe.2025.1600020

**Published:** 2025-07-09

**Authors:** Yong-Wang Zhang, Pei-Yu Du, Xi Li, Lu Liu, Yun-Tao Yan, Yi-Cong Bai, Xin-Yu Tian, Shuang-Qing Du

**Affiliations:** ^1^ Department of Orthopaedics, Hebei Provincial Hospital of Traditional Chinese Medicine, Shijiazhuang, China; ^2^ Department of Orthopaedics, The Third Hospital of Hebei Medical University, Shijiazhuang, China; ^3^ Preventive Treatment of Disease, Hebei Provincial Hospital of Traditional Chinese Medicine, Shijiazhuang, China

**Keywords:** knee osteoarthritis, coronal lumbar spine sequence, lower limb force line, waist and knee offset distance, correlation

## Abstract

**Objective:**

The aim of this study is to explore the correlation between the horizontal deviation of the coronal lumbar spine and the lower limb force line in knee osteoarthritis (KOA).

**Methods:**

A retrospective analysis of 233 KOA cases (47 male patients and 186 female patients, aged 50–83 years, with an average of 61.55 ± 8.72 years) admitted from 1 October 2022 to 31 December 2023 was conducted. Bilateral hip, knee, and ankle (HKA) angles; mechanical axis offset distance (MAD); joint line convergence angle (JLCA); lumbar–knee offset distance (LKOD, a self-tested angle reflecting the difference in the position of the bilateral knee joint relative to the lumbar vertebra); Western Ontario and McMaster Universities Arthritis Index (WOMAC) score; and baseline data records were assessed for all patients.

**Results:**

The WOMAC score was correlated with L1, L2, and L3 in LKOD (*P* < 0.05; r = 0.240, 0.362, and 0.386) but not with L4 and L5 (*P* > 0.05). WOMAC was also associated with HKA, MAD, and JLCA on the affected side (*P* < 0.05; r = −0.127, 0.140, and 0.135). The unaffected side and d-values were not associated with HKA, MAD, and JLCA (the d-value represents the absolute value of the difference between the unaffected and affected sides, which represents the overall change in both lower limbs) (*P* > 0.05). L1–L5 in LKOD was associated with HKA and MAD d-values (*P* < 0.05); LKOD was not correlated with HKA, MAD, and JLCA on the affected side.

**Conclusion:**

Lumbar horizontal deviation is a risk factor affecting all angles in both lower limbs and may aggravate knee bone and joint symptoms. The influence of horizontal direction changes in the lumbar spine on KOA, especially change in L3, should be prioritized in the clinical treatment and research of KOA.

## Introduction

Knee osteoarthritis (KOA) is a degenerative joint disease characterized by progressive hyaline cartilage destruction, sclerosis of the subchondral bone, and synovial inflammation. The main symptoms of KOA are knee pain and swelling (Sharma, 2021). KOA accounts for almost four-fifths of the burden of osteoarthritis (OA) worldwide and increases with age; the global incidence of KOA in individuals over 60 years is approximately 10% in men and 18% in women (Katz et al., 2021). Understanding the incidence, prevalence, and modifiable risk factors of KOA is necessary for developing effective preventive strategies.

As the hub of human weight bearing and movement, the horizontal shift may also affect the lower limb force line. Previous studies have evaluated spinal and pelvic parameters and knee flexion angles and have found that knee flexion is closely related to a compensatory mechanism and the reduction in lumbar lordosis angle in the sagittal plane (Tian et al., 2022; Obeid et al., 2011). Chen et al. analyzed the characteristics of sagittal morphology between patients with KOA and the normal population and discovered a sagittal imbalance in KOA patients, which may aggravate the degenerative changes in the lumbar spine (Weng et al., 2015). This study further explored the correlation between the horizontal deviation of the coronal lumbar spine and the lower limb force line in KOA. These data may provide a new theoretical basis for preventing and treating KOA.

## Methods

### Subjects

This study collected 286 patients with KOA from October 2022 to December 2023 in Orthopedics Clinic of Hebei Province Hospital of Traditional Chinese Medicine, and 233 patients were enrolled according to inclusion and exclusion criteria. The inclusion criteria were as follows: (1) patients diagnosed with KOA based on standard criteria (National Institute for Health and Care Excellence: Guidelines, 2022): (i) repeated knee pain within the past month; (ii) age ≥50 years; (iii) pain during standing or weight-bearing; X-ray shows joint space narrowing, subchondral bone sclerosis and (or) cystic change, and osteophyte formation at the articular edge; (iv) morning stiffness lasting 30 min; and (v) movement associated with bone friction tone (sensation), and KOA was diagnosed if criterion (i) and any of the other criteria (ii–v) were met; (2) diagnosis consistent with Kellgren–Lawrence (K–L) classification imaging criteria, using full weight-bearing radiographs of both lower limbs and lumbar X-ray, and K–L ≥ Ⅱ grade was considered (Kellgren, 1957); (3) consistent with unilateral knee joint incidence; (4) complete medical data available; and (5) no use of other drugs or external treatment within at least 2 weeks before testing. Exclusion criteria were as follows: (1) patients with severe osteoporosis, defined as a T-value ≤ −2.5 measured by lumbar DXA (Camacho et al., 2020); (2) postoperative patients who had undergone knee osteotomy or joint replacement; (3) patients with severe heart, brain, and kidney organic lesions or those experiencing active mental illness; and (4) patients with bone tumors or bone tuberculosis.

Demographic information, including age and gender, was extracted from the patient case records. Western Ontario and McMaster Universities Arthritis Index (WOMAC) scores and associated force angles were collected for all patients.

This study was conducted according to the Declaration of Helsinki (1964), and the protocol was approved by the hospital’s ethics committee [protocol number HBZY2022-KY-06 8-0168-01]. All patients voluntarily participated in this study and signed informed consent.

### Observational indicators

WOMAC score: a WOMAC scale with a total of 24 items and with individual scoring from 0 to 4 points was used to evaluate the structure and function of the knee joint from several aspects, such as pain, stiffness, and joint function status (Copsey et al., 2019).

Bilateral hip, knee, and ankle (HKA) angle: the angle formed between the line connecting the center point of the femoral head and the midpoint of the femoral socket and the line passing through the center point of the talus. Mechanical axis offset distance (MAD): the distance between the mechanical axis of the lower extremities (HKA) and the midpoint of the tibial intercondylar eminence. Joint line convergence angle (JLCA): the angle between the distal femoral joint line and the joint line of the tibial plateau (Meng et al., 2022).

Lumbar–knee offset distance (LKOD, clinical self-fitting angle): the midpoint of each vertebra from L1 to L5 was identified, and vertical lines were extended downward from these midpoints to assess their alignment relative to the knee joints. The distance of the medial femoral condyle of both knees to these plumb lines was measured. The difference between the distance between the knee joint and the midpoint of the same lumbar vertebral body was calculated, and the absolute value was also calculated. This absolute value can reflect the difference in the position of the two knee joints relative to the lumbar vertebra.

**FIGURE 1:**
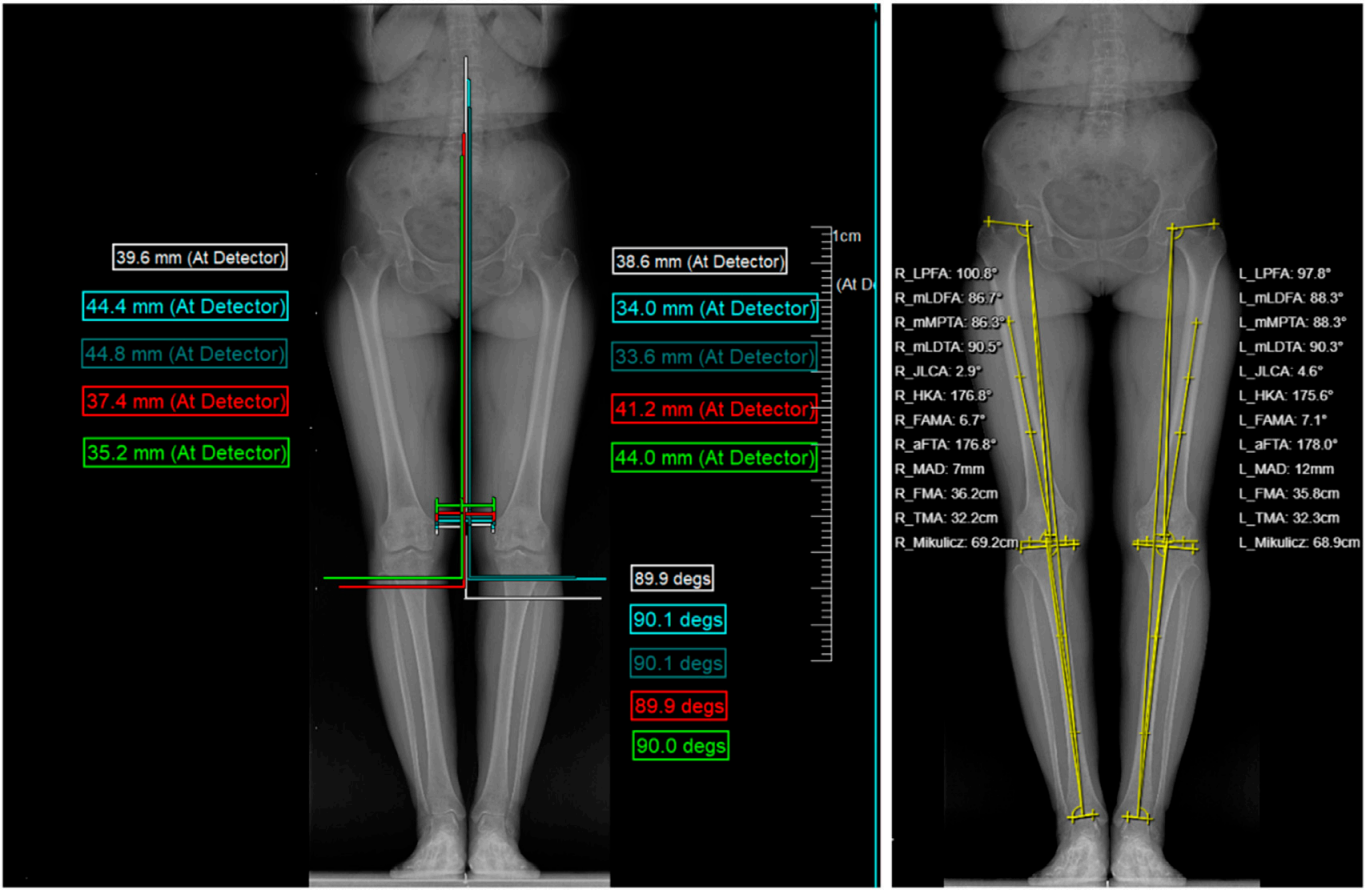
Abovementioned indicators record the unaffected side, affected side, and d-value (the absolute value of unaffected side and affected side difference).

The abovementioned angle is shown in Figure 1.

### Photography method display

**FIGURE 2:**
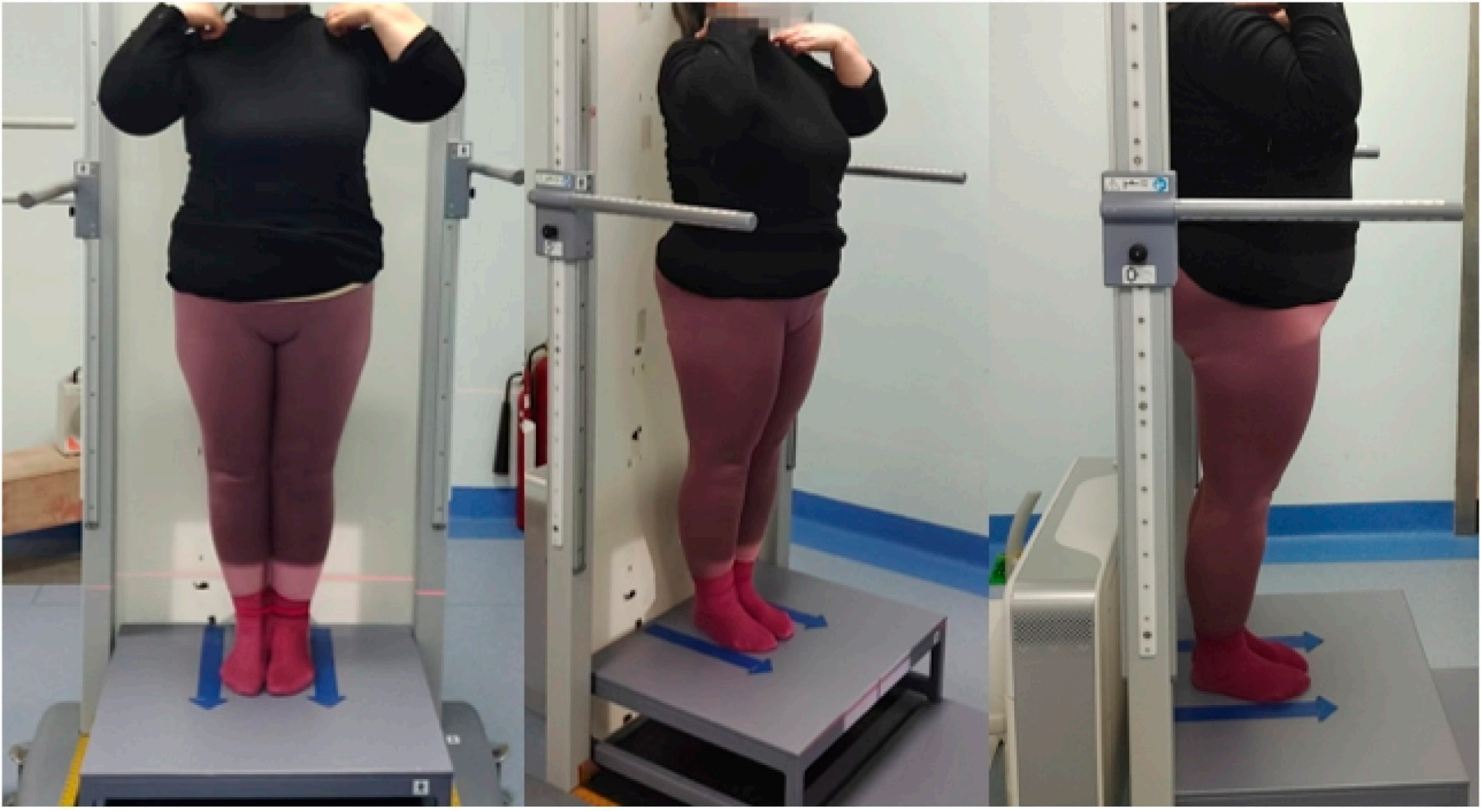
Camera angle (patient's front, patient's 45° orientation, patient's side).

A full-length, weight-bearing X-ray of the lumbar spine and both lower limbs was performed. An Optima XR646 HD digital medical X-ray system was used. The examination bed was adjusted to the standing vertical position, and the standing support plate was adjusted to the highest movable position (approximately 35 cm from the ground). The distance between the ball tube focus and the detector was 100 cm; the exposure range encompassed the upper edge, including the L1 vertebra, and the lower edge, including the ankle joint. The patient stood upright on the back of the photographic bed, facing the ball tube, placing the hands on both sides of the stent, the feet together, the patella and the toe forward, the lower limbs straight, and the posterior edge close to the photographic bed. Using panoramic photography, the ball tube was automatically exposed from top to bottom, while the detector moved with the ball tube from top to bottom, and the continuous exposure computer automatically generated the full-length weight-bearing position + lumbar X-ray image. Compared with the natural position, this fixed position can reduce the measurement error for subsequent studies, which is beneficial in comparing the change in the lower limb force line angle before and after treatment. The subject position is shown in Figure 2.

**FIGURE 3:**
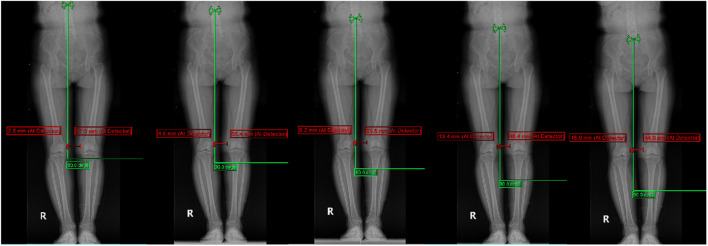
Patient 1: LKOD (L1: 54.2 mm, L2: 50.6 mm, L3: 43.4 mm, L4: 33 mm, L5: 28 mm).

**FIGURE 4:**
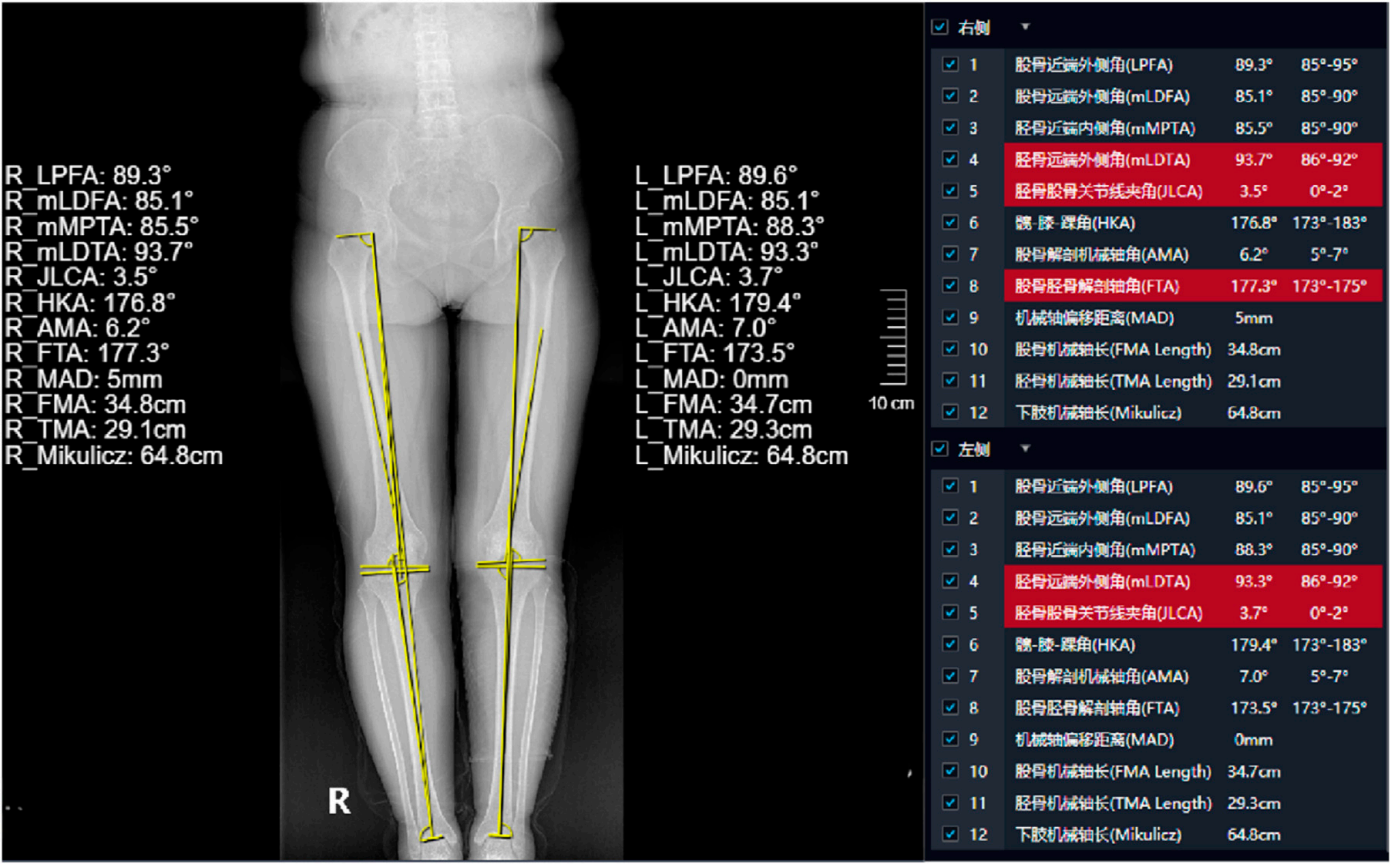
Patient 1: LKOD (L1: 54.2 mm, L2: 50.6 mm, L3: 43.4 mm, L4: 33 mm, L5: 28 mm).

**FIGURE 5:**
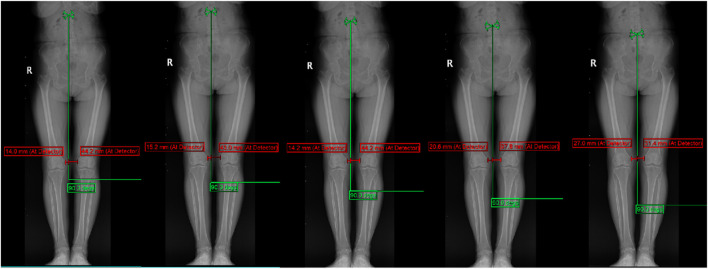
Patient 2: LKOD: (L1: 30.2 mm, L2: 27.8 mm, L3: 30 mm, L4: 17.2 mm, L5: 4.4 mm).

**FIGURE 6:**
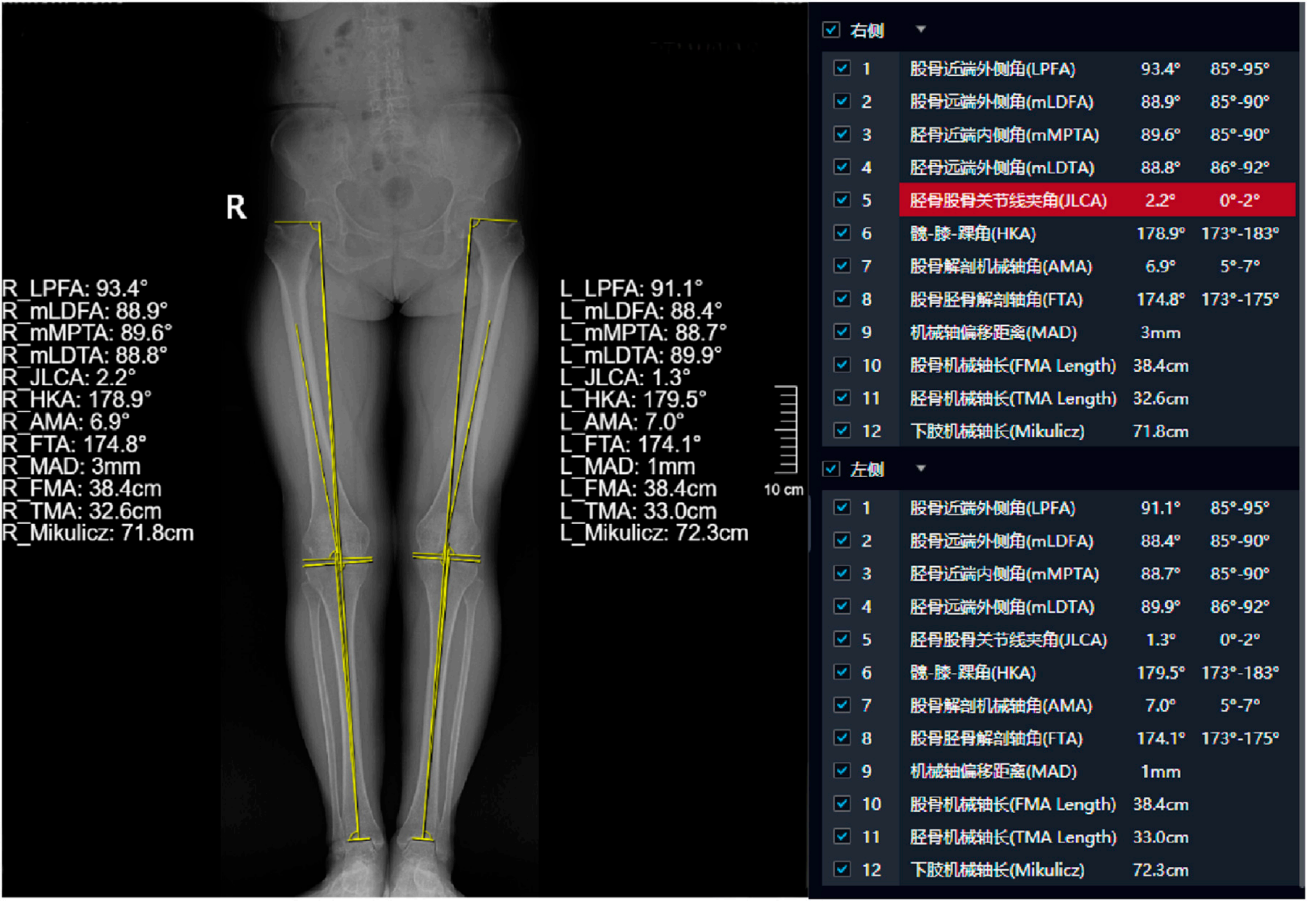
Patient 2: HKA (left: 179.5°, right: 178.9°, d-value: 0.6°), MAD (left: 1mm, right: 3mm, d-value: 2mm), JLCA (left: 1.3°, right: 2.2°, d-value: 0.9°).

Display of typical patient measurement data Patient 1: Gender: Female, age 50, WOMAC: 40 points.L1-L5 measurements are presented in Figure 3. The HKA, MAD, and JLCA measurements are presented in Figure 4. Patient 2: Gender: Female, age 56, WOMAC: 28 points.L1-L5 measurements are presented in Figure 5. The HKA, MAD, and JLCA measurements are presented in Figure 6.

### Measurement methods

All images were measured using MI platform software with a ruler and angle measurement tool. The same physician and technician measured all data three consecutive times, and the average value was taken as the final result.

### Statistical analysis

SPSS 26.0 statistical analysis software was used. Measurement data were described as the mean ± standard deviation (x ± s). The data were tested for normality. Pearson’s correlation test was used for normally distributed data, while Spearman’s correlation test was used for non-normally distributed data. Count data were expressed as frequencies (n). The chi-square test was used for correlation analysis. P < 0.05 was considered statistically significant.

## Results

A total of 233 KOA patients were included in this study, including 47 male patients and 186 female patients. The K–L classification was as follows: 92 cases of grade Ⅱ, 101 cases of grade Ⅲ, and 40 cases of grade Ⅳ. More general data are shown in Table 1.

**TABLE 1:** General characteristics of the study population ( $\bar{x}$ ± s).

Observational indicator	Siding-to-siding block	Mean ± standard deviation
Age (year)	50∼83	61.55 ± 8.72
Disease course (year)	0.25∼8	3.55 ± 3.41
BMI (kg/㎡)	18.83∼33.69	25.5 ± 3.28
Lumbar bone mineral density (g/cm^2^)	−2.3∼2.4	−1.27 ± 1.25
LKOD (mm)	0∼97.7	19.5 ± 17.3
MAD (mm)	−21∼57	10.5 ± 13.9
HKA (linear measure)	161.8∼179.4°	176.3 ± 4.42
JLCA (linear measure)	0∼12.4°	2.65 ± 1.77

Table 2 shows the correlation analysis between WOMAC and HKA, MAD, and JLCA-affected side, unaffected side, and d-values. Spearman correlation analysis showed that WOMAC was associated with HKA, MAD, and JLCA (P = 0.053, 0.0.032, and 0.039, respectively; r = −0.127, 0.140, and 0.135, respectively). WOMAC showed no correlation with HKA, MAD, JLCA, and d-values (*P* > 0.05).

**TABLE 2:** Correlation between WOMAC and HKA, MAD, and JLCA-affected side, unaffected side, and d-values.

Analysis group	Statistical metric	HKA	MAD	JLCA
Affected side	r	−0.127	0.140^a^	0.135^a^
	*p*	0.053	0.032	0.039
Unaffected side	r	−0.077	0.103	0.018
	*p*	0.241	0.118	0.784
d-value	r	0.112	0.103	0.118
	*p*	0.088	0.118	0.072

^a^ At the 0.05 level (two-tailed), the correlation is significant.

Table 3 shows the correlation analysis between WOMAC and corresponding vertebrae in LKOD. Spearman correlation analysis showed that WOMAC was correlated with L1, L2, and L3 in LKOD (P = 0.033, 0.004, and 0.013, respectively; r = 0.240, 0.362, and 0.386, respectively) but not with L4 and L5 (*P* > 0.05).

**TABLE 3:** Correlation between WOMAC and L1–L5 in LKOD.

Statistical metric	L1	L2	L3	L4	L5
r	0.240^a^	0.362^b^	0.386^a^	0.121	0.123
*p*	0.033	0.004	0.013	0.066	0.061

^a^ At the 0.05 level (two-tailed), the correlation is significant.

^b^ At the 0.01 level (two-tailed), the correlation was significant.

Table 4 shows the correlation analysis between the corresponding vertebral bodies and HKA, MAD, and JLCA in LKOD. L1–L5 in LKOD was positively correlated with HKA and MAD d-values (*P* < 0.05); L3 had the most robust positive correlations with HKA, MAD, and JLCA (P = 0.000, 0.000, and 0.046, respectively; r = 0.436, 0.427, and 0.231, respectively). There was no correlation between LKOD and HKA, MAD, or JLCA on either the unaffected or affected side.

**TABLE 4:** Correlations between the corresponding vertebral bodies in LKOD and the affected side, unaffected side, and d-values of HKA, MAD, and JLCA.

Group	Statistical metric	Analysis group	L1	L2	L3	L4	L5
HKA	Affected side	r	0.030	0.024	0.029	−0.042	−0.014
		*p*	0.651	0.718	0.664	0.522	0.837
	Unaffected side	r	−0.004	0.004	0.001	−0.098	−0.062
		*p*	0.952	0.949	0.982	0.136	0.344
	d-value	r	0.112	0.306^a^	0.436^a^	0.223^a^	0.202^a^
		*p*	0.058	0.002	0.000	0.001	0.002
MAD	Affected side	r	−0.018	−0.010	−0.017	0.043	0.014
		*p*	0.787	0.877	0.798	0.514	0.833
	Unaffected side	r	0.019	0.013	0.015	0.116	0.046
		*p*	0.776	0.842	0.821	0.078	0.480
	d-value	r	0.136^b^	0.304^a^	0.427^a^	0.220^a^	0.217^a^
		*p*	0.038	0.002	0.000	0.001	0.001
JLCA	Affected side	r	0.050	0.024	0.065	0.098	0.070
		*p*	0.450	0.716	0.325	0.137	0.287
	Unaffected side	r	0.078	0.043	0.058	0.117	0.086
		*p*	0.238	0.516	0.380	0.075	0.193
	d-value	r	0.090	0.123	0.231^b^	0.097	0.075
		*p*	0.171	0.060	0.046	0.138	0.252

^a^ At the 0.05 level (two-tailed), the correlation is significant.

^b^ At the 0.01 level (two-tailed), the correlation was significant.

## Discussion

KOA is a degenerative disease characterized by knee degeneration, often accompanied by degeneration in the lumbar spine, pelvis, and other joints. Conditions such as hyperplasia and scoliosis fall under the category of lumbar degeneration.

The lumbar spine affects the knee joint. Related studies have reported an increase in the spine tilt angle, head tilt, and sagittal imbalance in older individuals. These patients maintain the sagittal plane balance through the hip and knee joint flexion, and when the knee flexion exceeds the normal limit, this may lead to KOA. Moreover, in older individuals who develop KOA first, knee flexion contracture and forward center of gravity, increasing stress concentration of the lumbar facet process, the sagittal imbalance of the spine, and a series of pathological changes in the spine are likely to occur (Tauchi et al., 2015). Close mechanical relationships exist between the spine, pelvis, and lower limbs, which has important implications for understanding human posture, movement, and related health issues. The force line focuses on the transmission and distribution of force from the spine to the pelvis to the lower limbs and the interaction between the three. For example, the bending and rotation of the spine can affect the position and stability of the pelvis, while the tilt or rotation of the pelvis may affect the mechanical relationship of the lower limbs.

This study measured the WOMAC score, LKOD of the lumbar spine (L1–L5), and the angle of the lower limbs to assess the relationship between the morphological changes in the lumbar spine and the knee arthritis index and the angle of the lower limbs. Our data suggest that WOMAC is negatively correlated with HKA on the affected side, with a positive correlation; the smaller the WOMAC score, the smaller HKA, MAD, and JLCA, indicating that the degree of deformity increased. In addition, WOMAC was positively correlated with L1, L2, and L3 in LKOD but not with L4 and L5. Next, when the distance of the lumbar spine offset increased, the bilateral knee force and the degree of knee flexion changed, aggravating the patient’s symptoms. Moreover, compared with the influence of HKA, MAD, and JLCA on the WOMAC score index, LKOD was slightly higher than those of these three parameters. LKOD has a slight advantage over HKA, MAD, and JLCA in improving the WOMAC score index, but this can only be considered based on specific research evidence.

L1-5 in LKOD showed significant correlations with d value of HKA and MAD (P < 0.05), and L3 had the highest correlation with d value of JLCA (P < 0.05, r = 0.231), while no other vertebrae showed significant associations. LKOD had no correlation with HKA, MAD, and JLCA on the affected side. When defining LKOD, the value change was related to both sides; the spine–pelvis–lower limb force line is used to change the entire human lumbar spine, pelvis, and lower limbs. The d-value is the absolute value of the affected side difference; the correlation analysis of HKA, MAD, JLCA, and LKOD can better reflect the overall change in human mechanics.

In 2002, Tsuji et al. (Kechagias and Grivas, 2024) proposed the concept of “knee-spine syndrome,” suggesting that the change in the spine sequence (the reduction in the lordotic angle and sacral tilt angle of the lumbar spine) leads to increased tension in the thigh muscle when in the standing position and the forced knee flexion caused by low-back pain and patellofemoral arthralgia. Recent studies have provided increasing evidence linking trunk and hip kinematics to knee valgus mechanics. Lumbar and hip stability prevent knee abduction movement during walking (Tsuji et al., 2002; Powers, 2010; Hewett and Myer, 2011). LKOD changes the force of the internal and external compartments of the knee joint, and this result affects HKA, MAD, and JLCA.

We also found that L3 in LKOD was more associated with the WOMAC score, HKA, MAD, and JLCA than other vertebral bodies. However, JLCA was only associated with L3. Nakatsuji et al. (2022) analyzed the parameters related to the lumbar disc height and knee using X-rays, finding that the relationship between L2/L3 disc height and the medial knee space was particularly strong. The third lumbar vertebra is located in the center of the five lumbar vertebrae, which is the activity center of the lumbar spine movement. It has an extensive range of activity, the longest transverse process, and the starting point of the quadratus and psoas muscles. At the same time, the deep fascia of the transverse abdominal fascia and the latissimus dorsi muscle are attached to this area and are known to exert the greatest force. The nerve root in the waist is mainly responsible for controlling the muscle activity of the knee joint and nearby areas, while L2 nerve root mainly innervates the skin sensation of the front and anterior side of the thigh, as well as the hip flexion and the contraction of the adductor muscles. The L3 nerve root extends further downward, controlling the skin sensation of the front, anterior, and medial side of the thigh and controlling the contraction movement of the quadriceps and adductor muscles. One study examining the patient’s gait found that when the core muscle group muscle strength is weak, it increases knee pain and affects walking or standing in patients with KOA (Uehara et al., 2019; Ag Daud et al., 2023). The altered lumbar stability causes tension and imbalance in the associated muscle groups, especially the lumbar and hip muscles. This imbalance in muscle strength may affect the stability and stress situation of the knee joint, making the knee joint more vulnerable to injury.

The present study has some limitations. Although this study provides new insights into imaging relevance, some limitations should be acknowledged. First, our analysis focused primarily on establishing radiology-based correlations and, therefore, did not systematically assess short-term or medium-to-long-term clinical efficacy endpoints. The result of this paper is a new scientific hypothesis to explore the mechanism of LKOD malformation. The follow-up in-depth study of this study will be carried out soon, and preliminary experiments are being conducted in combination with clinical short- and long-term efficacy and other indicators.

To sum up, LKOD is related to the d-value of HKA, MAD, and JLCA, with L3 being the most correlated factor; LKOD is also a risk factor for the aggravation of knee joint symptoms. Therefore, in the clinical treatment and research of knee osteoarthritis, priority should be given to the influence of changes in the lumbar horizontal direction on knee osteoarthritis, especially at L3.

## Abbreviations

HKA, hip, knee, and ankle; MAD, mechanical axis offset distance; JLCA, joint line convergence angle; LKOD, waist–knee offset distance (self-tested angle, reflecting the difference in the position of the bilateral knee joint relative to this lumbar vertebra); WOMAC, Western Ontario and McMaster Universities Arthritis Index.

## References

[B1] Ag Daud D. M. Liau S. N. Sudi S. Mohd Noh M. Khin N. Y. (2023). A case report on core muscles training for knee osteoarthritis through core muscles activations and gait analysis. Cureus 15, e33918. 10.7759/cureus.33918 36819380 PMC9936829

[B2] Camacho P. M. Petak S. M. Binkley N. Diab D. L. Eldeiry L. S. Farooki A. (2020). American association of clinical endocrinologists/american college of endocrinology clinical practice guidelines for the diagnosis and treatment of postmenopausal osteoporosis-2020 update. Endocr. Pract. 26, 1–46. 10.4158/gl-2020-0524suppl 32427503

[B3] Copsey B. Thompson J. Y. Vadher K. Ali U. Dutton S. J. Fitzpatrick R. (2019). Problems persist in reporting of methods and results for the WOMAC measure in hip and knee osteoarthritis trials. Qual. Life Res. 28, 335–343. 10.1007/s11136-018-1978-1 30229533 PMC6373321

[B4] Hewett T. E. Myer G. D. (2011). The mechanistic connection between the trunk, hip, knee, and anterior cruciate ligament injury. Exerc Sport Sci. Rev. 39, 161–166. 10.1097/JES.0b013e3182297439 21799427 PMC4168968

[B5] Katz J. N. Arant K. R. Loeser R. F. (2021). Diagnosis and treatment of hip and knee osteoarthritis: a review. Jama 325, 568–578. 10.1001/jama.2020.22171 33560326 PMC8225295

[B6] Kechagias V. A. Grivas T. B. (2024). Hip-spine and knee-spine syndrome: is low back pain improved after total hip and knee arthroplasty? Cureus 16, e57765. 10.7759/cureus.57765 38716012 PMC11075773

[B7] Kellgren J. H. (1957). Lawrence JS: radiological assessment of osteo-arthrosis. Ann. Rheum. Dis. 16, 494–502. 10.1136/ard.16.4.494 13498604 PMC1006995

[B8] Meng X. Wang Z. Ma X. Liu X. Ji H. Cheng J. Z. (2022). Fully automated measurement on coronal alignment of lower limbs using deep convolutional neural networks on radiographic images. BMC Musculoskelet. Disord. 23, 869. 10.1186/s12891-022-05818-4 36115981 PMC9482267

[B9] Nakatsuji S. Kawada M. Takeshita Y. Matsuzawa Y. Hata K. Araki S. (2022). Effect of unilateral knee extension restriction on the lumbar region during gait. J. Healthc. Eng. 2022, 1–8. 10.1155/2022/1151753 PMC9424019 36046010

[B10] National Institute for Health and Care Excellence: Guidelines (2022). Osteoarthritis in over 16s: diagnosis and management. London: National Institute for Health and Care Excellence (NICE) Copyright © NICE. 36745715

[B11] Obeid I. Hauger O. Aunoble S. Bourghli A. Pellet N. (2011). Global analysis of sagittal spinal alignment in major deformities: correlation between lack of lumbar lordosis and flexion of the knee. Eur. Spine J. 20 (5), 681–685. 10.1007/s00586-011-1936-x 21870096 PMC3175917

[B12] Powers C. M. (2010). The influence of abnormal hip mechanics on knee injury: a biomechanical perspective. J. Orthop. Sports Phys. Ther. 40, 42–51. 10.2519/jospt.2010.3337 20118526

[B13] Sharma L. (2021). Osteoarthritis of the knee. N. Engl. J. Med. 384, 51–59. 10.1056/NEJMcp1903768 33406330

[B14] Tauchi R. Imagama S. Muramoto A. Tsuboi M. Ishiguro N. Hasegawa Y. (2015). Influence of spinal imbalance on knee osteoarthritis in community-living elderly adults. Nagoya J. Med. Sci. 77, 329–337. 26412878 PMC4574319

[B15] Tian G. Wang L. Liu L. Zhang Y. Zuo L. Li J. (2022). Kinematic alignment versus mechanical alignment in total knee arthroplasty: an up-to-date meta-analysis. J. Orthop. Surg. Hong. Kong 30, 10225536221125952. 10.1177/10225536221125952 36250421

[B16] Tsuji T. Matsuyama Y. Goto M. Yimin Y. Sato K. Hasegawa Y. (2002). Knee-spine syndrome: correlation between sacral inclination and patellofemoral joint pain. J. Orthop. Sci. 7, 519–523. 10.1007/s007760200092 12355123

[B17] Uehara K. Akai M. Doi T. Oka H. Iwaya T. (2019). Relationship between X-ray findings of lumbar spondylosis and knee pain. BMC Musculoskelet. Disord. 20, 379. 10.1186/s12891-019-2755-1 31421680 PMC6698333

[B18] Weng W. J. Wang W. J. Wu M. D. Xu Z. H. Xu L. L. Qiu Y. (2015). Characteristics of sagittal spine-pelvis-leg alignment in patients with severe hip osteoarthritis. Eur. Spine J. 24, 1228–1236. 10.1007/s00586-014-3700-5 25421550

